# Supplementary material for: Effects of rumen-bypass protein supplement on growth performance, hepatic mitochondrial protein complexes, and hepatic immune gene expression of beef steers with divergent residual feed intake
Source: PLoS One. 2024 Jul 3;19(7):e0293718. doi: 10.1371/journal.pone.0293718 (PMC11221652; doi:10.1371/journal.pone.0293718)
Supplement: Attachment — Submitted filename: Response to Reviewers .docx [file pone.0293718.s003.docx]

**RESPONSE TO REVIEWERS**

Editor’s comments

Your manuscript "Effects of rumen-bypass protein supplement on growth performance, hepatic mitochondrial protein complexes, and hepatic immune gene expression of beef steers with divergent residual feed intake" has been reviewed. Based on the feedback, substantial revisions are required:
Clarify and be consistent with terminology, specifically use "protein supplement" instead of "supplemental additive".

**AU**: **Supplemental additive has been updated as a protein supplement throughout the manuscript.**

Address discrepancies in P-values mentioned in the text and tables.

**AU: Thank you for catching that. We’ve updated that, find in line 225.**

Review and revise the content for accuracy and clarity, especially in lines 216-221 and 233-230. Include detailed and relevant information on the rumen-protected amino acid additive.
Define abbreviations at their first occurrence.

**AU: This has been corrected. Detailed and relevant information of the rumen bypass proteins, which is a commercial product, have been added. Lines 113 – 114.**

Ensure a consistent format throughout the manuscript, including decimal places in tables.
Adhere to the “Vancouver” citation style and reevaluate the number of references used.
Include key data such as amino acid concentrations or levels of rumen-degradable and undegradable protein, especially in tables.

**AU: Reference format has been updated to the “Vancouver” citation style.**

Provide a clear rationale for your choice of animal model and feeding practices.

**AU: This has been added based on the information of our previous study that demonstrated that amino acid metabolism is the most significant pathway associated with RFI variation in beef cattle. See lines 89 – 91.**

Reviewer #2: The manuscript describes the consequences of supplementation of rumen-bypass protein supplement or a rumen-protected amino acid additive. The research question is interesting, a novel and essential topic, but there are several concerns with the manuscript at this stage. I indicated my special comments in the docx file (CP MANUSCRIPT 10 Reviewed.docx). Unfortunately, these challenges combined would require a significant revision to the manuscript before it reached the standards of PLOS ONE.

Reviewer #1:

1. use "protein supplement" instead of "supplemental additive"

**AU**: **Supplemental additive has been updated as protein supplement throughout the manuscript.**

2. line 227: "there was a trend (P=0.09), however, in Table 3 we can see P=0.05?????
**AU: Thank you for catching that. We’ve updated that, find in line 231.**

3. line 216 - 221: Delete it.

**AU: Deleted**

4; line 233 - 230: Move it to line 216 and correct the P value according to Table 3 (P=0.05 instead of 0.09)

**AU: This has been corrected.**

5. line 347: Replace the sentence "Urea is........excretion by: The majority of urea in the bloodstream is eliminated via urine

**AU: I’ve updated text to “The majority of urea in the bloodstream is eliminated via urine”. See line 346**
